# Data Quality in Health Research: Integrative Literature Review

**DOI:** 10.2196/41446

**Published:** 2023-10-31

**Authors:** Filipe Andrade Bernardi, Domingos Alves, Nathalia Crepaldi, Diego Bettiol Yamada, Vinícius Costa Lima, Rui Rijo

**Affiliations:** 1 Ribeirão Preto School of Medicine University of Sao Paulo Ribeirão Preto Brazil; 2 Polytechnic Institute of Leiria Leiria Portugal; 3 Institute for Systems and Computers Engineering Coimbra Portugal; 4 Center for Research in Health Technologies and Services Porto Portugal

**Keywords:** data quality, research, digital health, review, decision-making, health data, research network, artificial intelligence, e-management, digital governance, reliability, database, health system, health services, health stakeholders

## Abstract

**Background:**

Decision-making and strategies to improve service delivery must be supported by reliable health data to generate consistent evidence on health status. The data quality management process must ensure the reliability of collected data. Consequently, various methodologies to improve the quality of services are applied in the health field. At the same time, scientific research is constantly evolving to improve data quality through better reproducibility and empowerment of researchers and offers patient groups tools for secured data sharing and privacy compliance.

**Objective:**

Through an integrative literature review, the aim of this work was to identify and evaluate digital health technology interventions designed to support the conducting of health research based on data quality.

**Methods:**

A search was conducted in 6 electronic scientific databases in January 2022: PubMed, SCOPUS, Web of Science, Institute of Electrical and Electronics Engineers Digital Library, Cumulative Index of Nursing and Allied Health Literature, and Latin American and Caribbean Health Sciences Literature. The Preferred Reporting Items for Systematic Reviews and Meta-Analyses checklist and flowchart were used to visualize the search strategy results in the databases.

**Results:**

After analyzing and extracting the outcomes of interest, 33 papers were included in the review. The studies covered the period of 2017-2021 and were conducted in 22 countries. Key findings revealed variability and a lack of consensus in assessing data quality domains and metrics. Data quality factors included the research environment, application time, and development steps. Strategies for improving data quality involved using business intelligence models, statistical analyses, data mining techniques, and qualitative approaches.

**Conclusions:**

The main barriers to health data quality are technical, motivational, economical, political, legal, ethical, organizational, human resources, and methodological. The data quality process and techniques, from precollection to gathering, postcollection, and analysis, are critical for the final result of a study or the quality of processes and decision-making in a health care organization. The findings highlight the need for standardized practices and collaborative efforts to enhance data quality in health research. Finally, context guides decisions regarding data quality strategies and techniques.

**International Registered Report Identifier (IRRID):**

RR2-10.1101/2022.05.31.22275804

## Introduction

In health care settings, the priceless value of data must be emphasized, and the relevance and performance of digital media are evidenced by the efforts of governments worldwide to develop infrastructure and technology, aiming to expand their ability to take advantage of generated data. It is important to emphasize that technology, by itself, cannot transform data into information, and the participation of health care professionals is essential for knowledge production from a set of data. Through research that optimizes health interventions and contributes to aligning more effective policies, knowledge combines concrete experiences, values, contexts, and insights, which may enable a framework for evaluation and decision-making [1].

The low quality, nonavailability, and lack of integration (fragmentation) of health data can be highlighted among the main factors that negatively influence research and health decision-making. In addition, it is worth noting the existence of a large number of remote databases accessible only in a particular context. Such factors cause data quality problems and, consequently, information loss. Despite the intense volume, information remains decentralized, but it needs to help the decision-making process [2], making its coordination and evaluation challenging.

The crucial role of data spans a wide range of areas and sectors, ranging from health care data to financial data, social media, transportation, scientific research, and e-commerce. Each data type presents its own challenges and requirements regarding quality, standardization, and privacy. Ensuring the quality and reliability of these data is essential to support the combination of different sources and types of data that can lead to even more powerful discoveries [3].

For example, using poor-quality data in developing artificial intelligence (AI) models can lead to decision-making processes with erroneous conclusions. AI systems, which are increasingly used to aid decision-making, have used labeled big data sets to build their models. Data are often collected and marked by poorly trained algorithms, and research often demonstrates this method’s problems. Algorithms can present biases in judgments about a person’s profession, nationality, or character and basic errors hidden in the data used to train and test their models. Consequently, prediction can be masked, making it difficult to distinguish between right and wrong models [4].

Principles are also established in the semantic web domain to ensure adequate data quality for use in linked data environments. Such recommendations are divided into 4 dimensions: quality of data sources, quality of raw data, quality of the semantic conversion, and quality of the linking process. The first principle is related to the availability, accessibility, and reliability of the data source, as well as technical issues, such as performance and verifiability [5]. The second dimension refers to the absence of noise, inconsistencies, and duplicates in the raw data from these data sources. In addition, it also addresses issues regarding the completeness, accuracy, cleanness, and formatting of the data to be helpful and easily converted into other models, if necessary. The last 2 dimensions refer to the use of high-quality validated vocabularies, flexible for semantic conversion, and the ability of these data to be combined with other semantic data, thus generating sophisticated informational intelligence. Such factors depend on correctness, granularity, consistency, connectedness, isomorphism, and directionality [6].

The heterogeneity of data in this area is intrinsically connected to the type of information generated by health services and research, which are considered diverse and complex. The highly heterogeneous and sometimes ambiguous nature of medical language and its constant evolution, the enormous amount of data constantly generated by process automation and the emergence of new technologies, and the need to process and analyze data for decision-making constitute the foundation for the inevitable computerization of health systems and research and to promote the production and management of knowledge [7].

There are different concepts of data quality [8]. According to the World Health Organization, quality data portray what was determined by their official source and must encompass the following characteristics: accuracy and validity, reliability, completeness, readability, timeliness and punctuality, accessibility, meaning or usefulness, confidentiality, and security [9]. Data quality can be affected at different stages, such as the collection process, coding, and nonstandardization of terms. It can be interfered with by technical, organizational, behavioral, and environmental aspects [10].

Even when data exist, some aspects make their use unfeasible by researchers, managers, and health care professionals, such as the noncomputerization of processes, heterogeneity, duplicity, and errors in collecting and processing data in health information systems [11]. Reliable health data must support decision-making and strategies to improve service delivery to generate consistent evidence on health status, so the data quality management process must ensure the reliability of the data collected [12].

Some health institutions have action protocols that require their departments to adopt quality improvement and resource-saving initiatives. Consequently, various methodologies to improve the quality of services have been applied in the health field. Mulgund et al [13] demonstrated, for example, how data quality from physician-rating sites can empower patients’ voices and increase the transparency of health care processes.

Research in scientific communities about new strategies constantly evolves to improve research quality through better reproducibility and empowerment of researchers and provides patient groups with tools for secure data sharing and privacy compliance [14]. Raising a hypothesis and defining a methodology are a standard scientific approach in health research, which will lead to the acquisition of specific data. In contrast, data production in the big data era is often completely independent of the possible use of the data. One of the hallmarks of the big data era is that the data are often used for a purpose other than the one for which they were acquired. In this sense, influencing the modification of acquisition processes in clinical contexts requires more structured approaches [13].

The health sector is increasingly using advanced technologies, such as sophisticated information systems, knowledge-based platforms, machine learning algorithms, semantic web applications, and AI software [15]. These mechanisms use structured data sets to identify patterns, resolve complex problems, assist with managerial and strategic decision-making, and predict future events. However, it is crucial to ensure that the data used for these analyses adhere to the best practices and metrics for evaluating data quality to avoid biases in the conclusions generated by these technologies. Failure to do so can make it challenging to elucidate previously unknown health phenomena and events [16].

To use the best practices, institutions use the results of literature reviews due to the significant time savings and high reliability of their studies. Thus, through an integrative literature review, the main objective of this work is to identify and evaluate digital health technology interventions designed to support the conduct of health research based on data quality.

## Methods

### Study Design

The Population, Concept, and Context (PCC) strategy was applied to define the research question. The PCC strategy guides the question of the study and its elaboration, helping in the process of bibliographic search for evidence. The adequate definition of the research question indicates the information necessary to answer it and avoids the error of unnecessary searches [17].

“Population” refers to the population or problem to be investigated in the study. “Content” refers to all the detailed elements relevant to what would be considered in a formal integrative review, such as interventions and phenomena of interest and outcomes. “Context” is defined according to the objective and the review question. It can be determined by cultural factors, such as geographic location, gender, or ethnicity [18]. For this study, the following were defined: P=digital technology, C=data accuracy, and C=health research.

In this sense, the following research questions were defined:

- What is the definition of health research data quality?
- What are the health research data quality techniques and tools?
- What are the indicators of the data confidence level in health research?

### Health Research

Numerous classifications characterize scientific research, depending on its objective, type of approach, and nature. Regardless of the purpose of how surveys can be classified, levels of confidence in data quality must be ubiquitous at all stages of the survey. Detailed cost-effectiveness analysis may inform decisions to adopt technology methods and tools that support electronic data collection of such interventions as an alternative to traditional methods.

Health research systems have invested heavily in research and development to support sound decisions. In this sense, all types of studies were observed that presented results of recent opportunities to apply the value of digital technology to the quality of the information in the direct or indirect evaluation of the promotion of health research. Therefore, in a transversal way, we considered all types of studies dealing with such aspects.

#### Types of Approaches

Various methods for setting priorities in health technology research and development have been proposed, and some have been used to identify priority areas for research. They include surveys and measurements of epidemiological estimates, clinical research, and cost-effectiveness assessments of devices and drugs. The technical challenges and estimation of losses due to variations in clinical practice and deviations from protocols have been supported by recommendation manuals and good practice guidelines. However, each of these proposed methods has specific severe methodological problems.

First, all these approaches see research simply as a method of changing clinical practice. However, there are many ways to change clinical practice, and conducting research may not be the most effective or cost-effective way. Research’s real value is generating information about what clinical practice should be. The question of how to implement survey results is a separate but related issue. Therefore, these methods implicitly assume no uncertainty surrounding the decision that the proposed research should inform.

#### Types of Interventions and Evaluated Results

Technology-based interventions that affect and aggregate concepts, designs, methods, processes, and outcomes promote data quality from all health research.

Measures demonstrate how results can address political, ethical, and legal issues, including the need to support and use technological mechanisms that bring added value regardless of the type and stage at which they are applied to research. We looked at how the results can be evaluated to address other questions, such as which subgroups of domains should be prioritized, which comparators and outcomes should be included, and which follow-up duration and moments would be most valuable for improving interventions on the reliability of health research data.

### Eligibility Criteria

Research carried out in English and Portuguese, with quantitative and qualitative approaches, primary studies, systematic reviews, meta-analyses, meta-synthesis, books, and guidelines, published from 2016 onward was included. This choice is justified because we sought scientific indications that were minimally evaluated by our community. In this sense, websites, white papers, reports, abstracts only, letters, and commentaries were not considered. The year limitation is justified because knowledge is considered an adequate degree of being up to date.

In addition to the methodological design, we included any studies that described the definition, techniques, or tools that have the essential functions of synthesis, integration, and verification of existing data from different research sources to guarantee acceptable levels of data quality. In this way, we expected to monitor trends in health research, highlight areas for action on this topic, and, finally, identify gaps in health data arising from quality control applications.

Although the primary objective of this review was to seek evidence of data quality from health research, we also independently included studies on health data quality and research data quality. The exclusion criteria were applied to studies with a lack of information (eg, the paper was not found), studies whose primary focus was not health and research, and papers not relevant to the objective of the research, papers not available as full text in the final search, and papers not written in English or Portuguese. In addition, the titles and respective authors were checked to verify possible database repetitions. All criteria are presented in Table 1.

**Table 1:** Inclusion and exclusion criteria for eligibility of studies.

Category	Inclusion criteria	Exclusion criteria
Approach	Quantitative, qualitative	—^a^
Document type	Primary studies, systematic reviews, meta-analyses, meta-synthesis, books, guidelines	Websites, white papers, reports, abstracts only, letters, commentaries
Year	Starting from 2016	Before 2016
Information	Describe the definition, techniques, or tools that have functions of synthesis, integration, and verification of existing data from different research sources	Lack information, not available as full text
Study focus	—	Not health and research, not relevant to the objective
Language	—	Not in English or Portuguese

^a^Not applicable.

### Databases and Search Strategies

A search was carried out in 6 electronic scientific databases in January 2022 because of their quality parameters and broad scope: PubMed, SCOPUS, Web of Science, Institute of Electrical and Electronics Engineers (IEEE) Digital Library, Cumulative Index of Nursing and Allied Health Literature (CINAHL), and Latin American and Caribbean Health Sciences Literature (LILACS). For the search, descriptors and their synonyms were combined according to the Health Sciences Descriptors (DeCS) [19] and Medical Subject Headings (MeSH) [20]. The following descriptors and keywords were selected, combined with the Boolean connectors AND and OR: “Data Accuracy,” “Data Gathering,” and “Health Research.” These descriptors and keywords come from an iterative and tuning process after an exploratory phase. The same search strategy was used in all databases.

Google Scholar was used for manual searching, searching for other references, and searching for dissertations. These documents are considered gray literature because they are not published in commercial media. However, they may thus reduce publication bias, increase reviews’ comprehensiveness and timeliness, and foster a balanced picture of available evidence [21].

We created a list of all the studies we found and removed duplicates. A manual search was performed for possible studies/reports not found in the databases. The references of each analyzed study were also reviewed for inclusion in the search. The search was carried out in January 2022, and based on the inclusion and exclusion criteria described, the final number of papers included in the proposed integrative review was reached. The search procedure in the databases and data platforms is described in Table 2, according to the combination of descriptors.

**Table 2:** Search procedure on databases.

Database	Search string	Query result (N=27,709), n (%)
PubMed	(“Data Accuracy” OR “Data Gathering”) AND “Health Research”	19,340 (69.80)
SCOPUS	TITLE-ABS-KEY ((data AND accuracy OR data AND gathering) AND health AND research) AND (LIMIT-TO (PUBYEAR , 2021) OR LIMIT-TO (PUBYEAR , 2020) OR LIMIT-TO (PUBYEAR , 2019) OR LIMIT-TO (PUBYEAR , 2018) OR LIMIT-TO (PUBYEAR , 2017) OR LIMIT-TO (PUBYEAR , 2016))	789 (2.84)
Web of Science	((“Data Accuracy” OR “Data Gathering”) AND “Health Research”)	5589 (20.17)
IEEE^a^ Digital Library	(“Index Terms”:Data Accuracy) OR (“Index Terms”:Data Gathering) AND (“Index Terms”:Health Research)	1989 (7.18)
LILACS^b^	Data Accuracy [Palavras] or Data Collection [Palavras] and Health Research Evaluation [Palavras]	2 (0.01)
CINAHL^c^	(“Data Accuracy” OR “Data Gathering”) AND “Health Research”	0

^a^IEEE: Institute of Electrical and Electronics Engineers.

^b^LILACS: Latin American and Caribbean Health Sciences Literature.

^c^CINAHL: Cumulative Index of Nursing and Allied Health Literature.

### Data Collection

First, 2 independent reviewers with expertise in information and data science performed a careful reading of the title of each paper. The selected papers were filtered after reading the abstract and selected according to the presence of keywords and descriptors of interest. The reviewers were not blinded to the journal’s title, study authors, or associated institutions. The established inclusion and exclusion criteria adequacy was verified for all screened publications. Any disagreements between the 2 reviewers were resolved by a senior third independent evaluator. The Mendeley reference manager [22] was used to organize the papers. Subsequently, the extracted findings were shared and discussed with the other team members.

Data synthesis aims to gather findings into themes/topics that represent, describe, and explain the phenomena under study. The extracted data were analyzed to identify themes arising from the data and facilitate the integration and development of the theory. Two reviewers performed data analysis and shared it with other team members to ensure the synthesis adequately reflected the original data.

### Data Extraction

Data extraction involved first-order (participants’ citations) or second-order (researchers’ interpretation, statements, assumptions, and ideas) concepts in qualitative research. Second-order concepts were extracted to answer the questions of this study [17].

We looked at data quality characteristics in the studies examined, the assessment methods used, and basic descriptive information, including the type of data under study. Before starting this analysis, we looked for preexisting data quality and governance models specific to health research but needed help finding them. Thus, 2 reviewers were responsible for extracting the following data from each paper:

- Bibliographic information (title, publication date and journal, and authors)
- Study objectives
- Methods (study design, data collection, and analysis)
- Results (researchers’ interpretation, statements, assumptions, and ideas)

### Result Presentation

The PRISMA (Preferred Reporting Items for Systematic Reviews and Meta-Analyses) checklist (Multimedia Appendix 1) and flowchart were used to visualize the search strategy results in the databases. PRISMA follows a minimum set of items to improve reviews and meta-analyses [23]. Based on the PRISMA flowchart, a narrative synthesis was prepared, in which we described the objectives and purposes of the selected and reviewed papers, the concepts adopted, and the results related to the theme of this review.

### Data Synthesis

The data synthesis process involved several steps to ensure a systematic and comprehensive analysis of the findings. After a rigorous study selection process, the extracted data were analyzed using a coding and categorization approach.

Initially, a coding framework was developed based on the research objectives and key themes identified in the literature. This framework served as a guide for organizing and categorizing the extracted data. At least 2 independent reviewers performed this coding process to ensure consistency and minimize bias. Any discrepancies or disagreements were resolved through consensus discussions. Relevant data points from each study were coded and assigned to specific categories or themes (Multimedia Appendix 2), capturing the main aspects related to data quality in health research, as shown in Table 3.

**Table 3:** Search procedure used on databases.

Category	Subtopics/example codes
Data quality assessment methods	Ontologies, adjust to fit, frameworks, guidelines Quality dimensions
Factors influencing data quality	Study design, application/data sources Context, limitations
Strategies for improving data quality	Process, tools, techniques/analysis

Once the data were coded and categorized, a thorough analysis was conducted to identify patterns, trends, and commonalities across the studies. Quantitative data, such as frequencies or percentages of reported data quality issues, were analyzed using descriptive statistics. Qualitative data, such as themes or explanations provided by the authors, were analyzed using thematic analysis techniques to identify recurring concepts or narratives related to data quality.

The synthesized findings were then summarized and organized into coherent themes or subtopics. This involved integrating the coded data from different studies to identify overarching patterns and relationships. Similar results were grouped, and relationships between different themes or categories were explored to derive meaningful insights and generate a comprehensive picture of data quality in health research.

As part of the data synthesis process, the quality of the included studies was also assessed. This involved evaluating the studies’ methodological rigor, reliability, and validity using established quality assessment tools or frameworks. The quality assessment results were considered when interpreting and discussing the synthesized findings, providing a context for understanding the strength and limitations of the evidence.

## Results

### Study Characteristics

In this review, 27,709 occurrences were returned from the search procedure, with 789 (2.84%) records from the SCOPUS database, 2 (0.01%) from LILACS, 1989 (7.18%) from the IEEE Digital Library, 5589 (20.17%) from the Web of Science, and 19,340 (69.80%) from PubMed. Searches were also performed in the World Health Organization Library and Information Networks for Knowledge (WHOLIS) and CINAHL databases, but no results were found. Of these, 25,202 (90.95%) records were flagged as ineligible by the automation tools and filters available in the databases, because they were mainly reports, editorial papers, letters or comments, book chapters, dissertations, and theses or because they did not specifically address the topic of interest according to the use of descriptors. Furthermore, 204 (0.74%) records were duplicated between databases and were removed.

**Figure 1:**
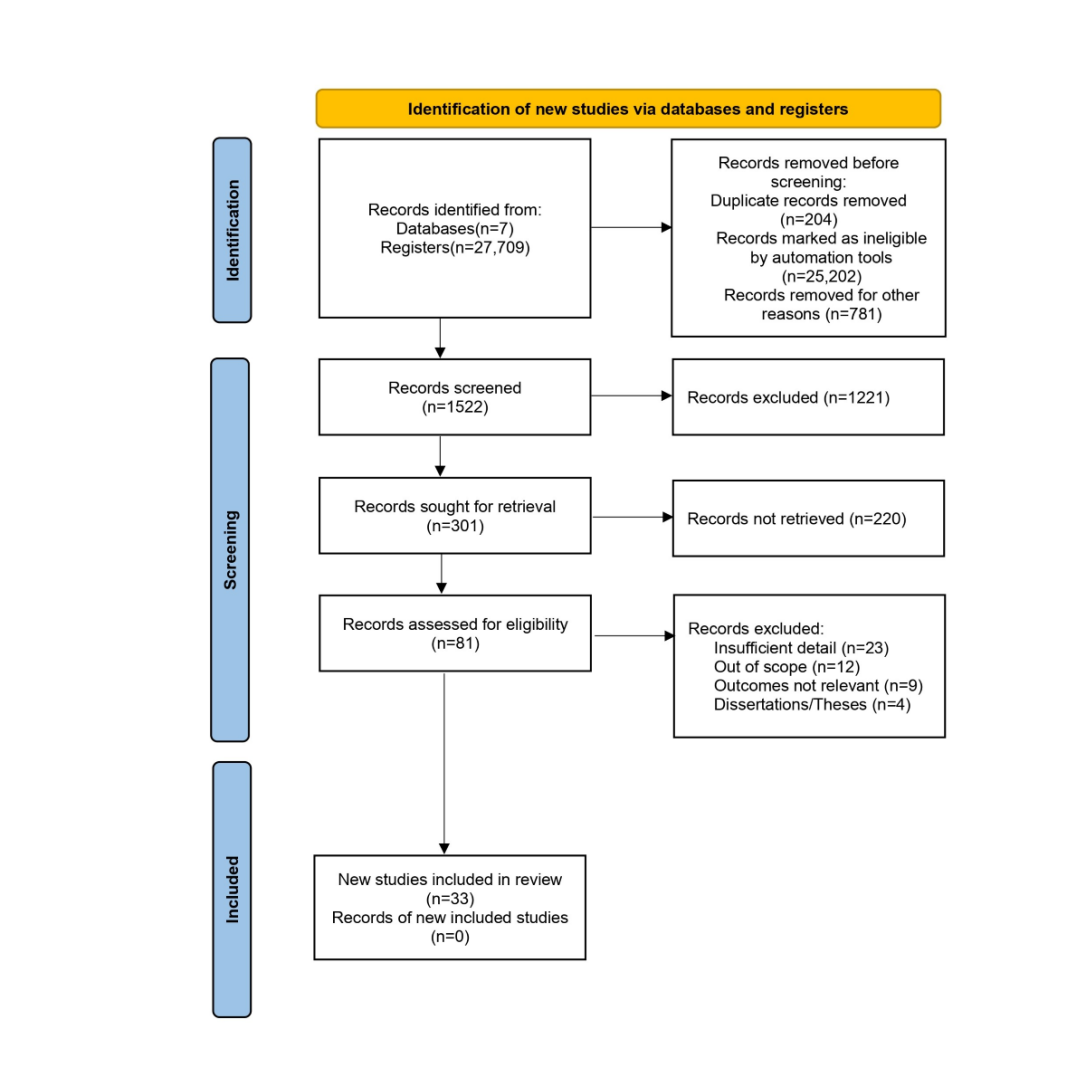
PRISMA flowchart with the results of study selection. PRISMA: Preferred Reporting Items for Systematic Reviews and Meta-Analyses.

After carefully evaluating the titles and abstracts (first screening step), 1221 (80.22%) of 1522 search results were excluded. For inclusion of papers after reading the abstracts, 81 () of 301 (26.9%) papers were listed for a full reading. After analyzing and extracting the desired results, 33 (40.7%) papers were included in the review because they answered the research questions. The entire selection, sorting, extraction, and synthesis process is described through the PRISMA flowchart [23], represented in Figure 1.

The 33 studies covered the period of 2017-2021 and were conducted in 22 countries. Most studies were concentrated in Europe (n=11, 33.3%) and North America (the United States and Canada; n=10, 30.3%). Others were carried out in Oceania (Australia; n=4, 12.1%), Asia (China and Taiwan; n=3, 9.1%), and the Middle East (Iran and Saudi Arabia; n=2, 6.1%). In addition, studies were carried out collaboratively or in a network (the United States and India; the United States and African countries; the US Consortium, the United Kingdom, South Africa, Costa Rica, Canada, Sweden, Switzerland, and Bahrain; n=3, 9.1%).

In their entirety, the studies were carried out in high-income countries, and most of the assessments were based on the evidence available in English. The United States (n=11, 33.3%) and Australia (n=4, 12.1%) led in studies involving the investigated topic. No studies conducted or coordinated by middle-income countries were reported. In addition to the low economic diversity of countries where the research was conducted, all papers were evaluated in a single language. The involvement and collaboration of emerging countries took place exclusively through partnerships and participation in consortia.

Regarding the domains described in the studies, there was tremendous variability and inconsistency between the terms presented (n=38 terms). Note that no consensus existed between critical and noncritical variables for data quality assessment. The lack of consensus reflected that the definitions of concepts vary and their relationships are not homogeneous across studies. The discrepancy between domains and evaluated concepts did not allow an evaluation of parity between metrics and was present during all phases of the studies found. The subtopic distribution into the defined categories also evidenced the high-variability factors and strategies in the literature to lead with data quality. The distribution of the improvement strategies for data quality is shown in Table 4 and that of the related influencing factors for data quality in Table 5.

**Table 4:** Improvement strategies for data quality.

Category and improvement strategy		Distribution of improvement strategy in studies (N=33), n (%)
**Process**		
	Business intelligence model	12 (36.4)
	Monitoring	7 (21.2)
	Benchmarking	3 (9.1)
	No well-established process followed	11 (33.3)
**Techniques/analysis**		
	Quantitative	12 (36.4)
	Qualitative	11 (33.3)
	Mixed	10 (30.3)
**Tools**		
	Minimum data set definition	7 (21.2)
	Audit	13 (39.4)
	Error detection	3 (9.1)
	Decision support	2 (6.1)
	Multiple tools	2 (6.1)
	No tools used	6 (18.2)

**Table 5:** Influencing factors for data quality.

Strategy and influencing factors		Distribution of influencing factor in studies (N=33), n (%)
**Strategy: process; influencing factor category: context**		
	Research-only controlled environment	7 (21.2)
	Transition and validation environment	8 (24.2)
	Restricted routine environment	18 (54.6)
**Strategy: techniques/analysis; influencing factor category: study design**		
	Longitudinal	27 (81.8)
	Cross-sectional	3 (9.1)
	Combined	2 (6.1)
	No information provided/design adopted unclear	1 (3.0)
**Strategy: tools; influencing factor category: data source**		
	Own research repositories	24 (72.7)
	Preexisting data models	4 (12.1)
	Public databases	2 (6.1)
	Other sources	3 (9.1)
**Strategy: tools; influencing factor category: study limitations**		
	Methodological	21 (63.6)
	Technical	15 (45.5)
	Social	6 (18.2)
	Organizational	2 (6.1)
	Legal	1 (3.0)
**Strategy: tools; influencing factor category: study design**		
	Longitudinal	27 (81.8)
	Cross-sectional	3 (9.1)
	Combined	2 (6.1)
	No information provided/design adopted unclear	1 (3.0)

### Data Quality Issues and Challenges

The metrics extracted from the studies comprised domains related to the methodology adopted by them, that is, concepts that supported the definition of data quality and their respective individual or combined categorizations regarding the adjusted use for the purpose (n=8, 24.2%) of frameworks (n=6, 18.2%), ontologies (n=2, 6.1%), good practice guides (n=15, 45.5%), or combinations of methodologies (n=2, 6.1%).

Among the studies that used the concept of purpose-adjusted use, terms such as “gold standard according to experts” [24], “intrinsic quality” [25], “ideal record” [26], “data fitness” [27,28], and “data culture” [29,30] were addressed. In general, the use of frameworks and ontologies was based on previously published studies and available in development libraries as modules for mapping-adapted entities, proprietary or embedded systems, and data-based strategies for process improvement [31-34].

The central guides and guidelines adopted in data quality studies refer to the adoption of national protocols and policies, agreements signed between research networks and consortia, guides to good clinical practices (International Conference on Harmonization—Good Clinical Practice, ICHGCP [35-38]; Food and Drug Administration, FDA [35,38]; Health Insurance Portability and Accountability Act, HIPPA [39]), or information governance principles, models, and strategies (International Organization for Standardization, ISO [40,41]; Joint Action Cross-Border Patient Registries Initiative, PARENT [41]; Findability, Accessibility, Interoperability, and Reuse, FAIR [25,40,42]).

Regarding data quality, dimensions were interposed in all research stages, thus being a fundamental factor in being incorporated with good practices and recommendations, giving light to health research, regardless of their methodological designs. The distribution of dimensions evaluated in our findings showed significant heterogeneity, as shown in Table 6.

**Table 6:** Distribution of quality dimensions in health research.

Data quality dimension(s)	Distribution in studies (N=33), n (%)
Integrity	19 (57.6)
Precision	16 (48.5)
Consistency	11 (33.3)
Opportunity	10 (30.3)
Validity, plausibility	7 (21.2)
Relevance	5 (15.1)
Accuracy, accessibility, utility, conformity	4 (12.1)
Reliability, trust, interoperability, usability	3 (9.1)
Correctness, comparability, inconsistency, flexibility, security, availability	2 (6.1)
Credibility, incompleteness, bias, variance, frequency, prevention, singularity, temporality, exclusivity, uniqueness, currentness, consent, loss, degradation, simplicity, acceptability, interpretability, coherence	1 (3.0)

### Factors Affecting Data Quality

The study considered factors such as the environment, application time, and development steps, all influencing data quality. Controlled environments were reported in research-only scenarios with planning and proof-of-concept development [34,35,37,38,43-45]. Transition and validation environments were identified where research and service were combined [25,27,31,40,46-49]. Most studies were conducted in restricted environments specific to health services. Most studies also used their own research repositories, while others relied on external sources, such as preexisting data models [25,26,33,40] or public databases [38,50]. The research applications spanned diverse health areas, including electronic health records, cancer, intensive care units, rare diseases, maternal health, and more. However, the research areas were more concentrated in specialties such as clinical research [27,31,35,37,48], health informatics [43,45], and research networks [25,34,40,44,49]. Collaborative research networks and clinical trials played a prominent role in the application areas.

Data sources used in the research included literature papers, institutional records, clinical documents, expert perceptions, data models, simulation models, and government databases. Technical limitations were related to performance concerns, infrastructure differences, security measures, visualization methods, and access to data sources.

Other aspects mentioned included the disparity in professionals’ knowledge, the inability to process large volumes of information, and the lack of human and material resources. Legal limitations were attributed to organizational policies that restricted extensive analysis.

The main challenge reported in the studies was related to methodological approaches, particularly the inability to evaluate solutions across multiple scopes, inadequate sample sizes, limited evaluation periods, the lack of a gold standard, and the need for validation and evaluation in different study designs.

Overall, the integrated findings highlight the importance of considering the environment, application time, and methodological approaches in ensuring data quality in health research. The identified challenges and limitations provide valuable insights for future research and the development of strategies to enhance data quality assurance in various health domains.

### Strategies for Improving Data Quality

In the analyzed studies, various strategies and interventions were used to plan, manage, and analyze the impact of implementing procedures on data quality assurance. Business intelligence models guided some studies, using extraction, transform, and load (ETL) [32,40,41,47,51]; preprocessing [28,45,52-54]; Six Sigma practices [32,48]; and the business process management (BPM) model [33]. Data monitoring strategies included risk-based approaches [36,37], data source verification [35,37,38], central monitoring [37,38], remote monitoring (eg, telephone contact) [31,38], and training [29]. Benchmarking strategies were applied across systems or projects in some cases [26,50,51].

Quantitative analyses primarily involved combined strategies, with data triangulation often paired with statistical analyses. Data mining techniques [24], deep learning, and natural language processing [45] were also used in combination or individually in different studies. Statistics alone was the most commonly used quantitative technique. The qualitative analysis encompassed diverse approaches, with consultation with specialists [30,34,43,44,54,55], structured instruments [29,38,44,46], data set validation [41,42,56], and visual analysis [33,40,48] being prominent. Various qualitative techniques, such as interviews [27], the Delphi technique [24], feedback audit [35], grammatical rules [39], and compliance enforcement [49], were reported.

Different computational resources were used for analysis and processes. The R language (R Core Team and the R Foundation for Statistical Computing) was commonly used for planning and defining data sets, while Python and Java were mentioned in specific cases for auditing databases and error detection. Clinical and administrative software, web portals, and electronic data capture platforms (eg, Research Electronic Data Capture [REDCap], CommonCarecom, MalariaCare, Assistance Publique–Hôpitaux de Paris–Clinical Data Repository [AP-HP-CDR], Intensive Care Unit DaMa–Clinical information System [ICU-DaMa-CIS]) were used for support, decision-making, data set planning, collection, and auditing. Additional tools, such as dictionaries, data plans, quality indicators, data monitoring plans, electronic measurements (e-measures), and Microsoft Excel spreadsheets, were also used.

It is evident that a range of strategies, interventions, and computational resources were used to ensure data quality in the studies. Business intelligence models, statistical analyses, data mining techniques, and qualitative approaches played significant roles in analyzing and managing data quality. Various programming languages and software tools were used for different tasks, while electronic data capture platforms facilitated data collection and auditing. The integration of these findings highlights the diverse approaches and resources used to address data quality in the analyzed studies.

### Synthesis of Findings

The main barriers reported related to the theme of research in the area of health data quality cite circumstances regarding use, systems, and health services. Such barriers are influenced by technical, organizational, behavioral, and environmental factors that cover significant contexts of information systems, specific knowledge, and multidisciplinary techniques [43]. The quality of each data element in the 9 categories can be assessed by checking its adherence to institutional norms or by comparing and validating it with external sources [41]. Table 7 summarizes the main types of obstacles reported in the studies.

**Table 7:** Barriers to health data quality.

Barrier	Examples
Technical	Restrictive data formats Lack of metadata and standards Absence of technical solutions (eg, interoperability) Poor design quality (standards), development (flexibility), and evaluation (usability and complexity) of system designs Lack of detailed information for specific searches Terminology variations Limited recovery capabilities A large amount of unstructured data Challenges with patient identification and matching
Motivational	Lack of incentives to use data in decision-making Lack of delegation of responsibilities
Economical	Lack of investments in people, infrastructure, and organizational processes for collecting, storing, analyzing, and sharing data
Political	Lack of confidence Absence of restrictive guidelines and policies Lack of clarity of role and data owners
Legal	Intellectual property Copyright Data privacy Interest conflicts
Ethical	Purpose of data use Impact on data holders
Organizational	Organizational culture Low dissemination of research activities
Human Resources	Inadequate number of qualified and motivated personnel Little or no supervision Heavy workload Team rotations
Methodological	Sample size Little or no training in data analysis and interpretation tools Data extraction issues Unfamiliarity with data quality assessment Source document complexity Study design Measured variables (primary or secondary) Data collection time Encoding methods Transcription errors

Although many electronic records provide a dictionary of data from their sources, units of measurement were often neglected and adopted outside of established standards. Such “human errors” are inevitable, reinforcing the need for continuous quality assessment from the beginning of collection. However, some studies have tried to develop ontologies to allow the automated and reproducible calculation of data quality measures, although this strategy did not have great acceptance. For Feder [55], “The harmonized data quality assessment terminology, although not comprehensive, covers common and important aspects of the quality assessment practice.” Therefore, generating a data dictionary with its determined types and creating a data management plan are fundamental in the planning of research [28].

Both the way of collecting and the way of inputting data impact the expected result from a data set. Therefore, with a focus on minimizing data entry errors as an essential control strategy for clinical research studies, implementing intervention modes of technical barriers was presented as pre- and postanalysis [56]. The problems were caused by errors in the data source, extraction, transform, and load process or by limitations of the data entry tool. Extracting information to identify actionable insights by mining clinical documents can help answer quantitative questions derived from structured health quality research data sources [39].

Given the time and effort involved in the iterative error detection process, typical manual curation was considered insufficient. The primary sources of error included human and technological errors [35]. However, outliers identified by automated algorithms should be considered potential outliers, leaving the field specialists in charge [51]. In contrast, different and ambiguous definitions of data quality and related characteristics in emergency medical services were presented [55]. Such divergences were based on intuition, previous experiences, and evaluation purposes. Using definitions based on ontology or standardization is suggested to compare research methods and their results. The definitions and relationships between the different data quality dimensions were unclear, making the quality of comparative assessment difficult [52].

In terms of evaluation methods, similar definitions overlapped. The difference lay in the distribution comparison and validity verification, where the definition of distribution comparison was based on comparing a data element with an official external reference [54]. Meanwhile, the validity check was concerned with whether a particular value wass an outlier, a value outside the normal range. The reasons for the existence of multiple evaluation practices were the heterogeneity of data sources about syntax (file format), schema (data structure models), and semantics (meaning and varied interpretations) [50]. There should be a standard set of data to deal with such inconsistencies and allow data transformation into a structure capable of interoperating with its electronic records [40].

Data standardization transforms databases from disparate sources into a standard format with shared specifications and structures. It also allows users from different institutions to share digital resources and can facilitate the merging of multicenter data and the development of federated research networks [34]. For this, 2 processes are necessary: (1) standardization of individual data elements, adhering to terminology specifications [49], and (2) standardization of the database structure through a minimum data set, which specifies where data values are located and stored in the database [50]. Improvements in electronic collection software functionality and its coding structures have also been reported to result in lower error rates [36].

In addition, it is recommended to know the study platform and access secondary data sources that can be used. In this way, transparency in the systemic dissemination of data quality with clear communication, well-defined processes, and instruments can improve the multidisciplinary cooperation that the area requires [44].

Awareness campaigns on the topic at the organizational level contributed to improving aspects of data governance. The most reported error prevention activities were the continuing education of professionals with regular training of data collectors during their studies [50]. In this sense, in-service education should promote the correct use of names formulated by structured systems to improve the consistency and accuracy of records and favor their regular auditing. Health systems that received financial incentives for their research obtained more satisfactory results regarding the degree of reliability of their data [53].

**Figure 2:**
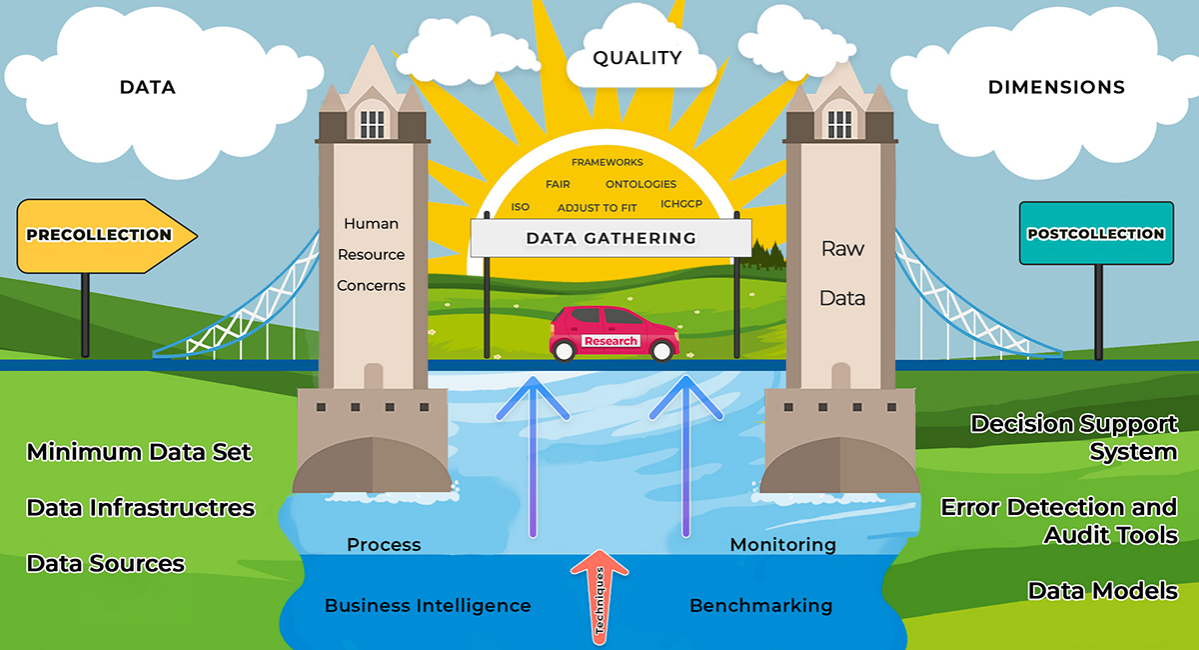
Elements involved in the research data quality process. Elements involved in the data quality process. FAIR: Findability, Accessibility, Interoperability, and Reuse; ICHGCP: International Conference on Harmonization—Good Clinical Practice; ISO: International Organization for Standardization.

Figure 2 depicts the great diversity of elements involved in the data quality process in health research, representing the planning (precollection), development (data acquisition and monitoring), and analysis (postcollection) stages. In our findings, each phase presented a set of strategies and tools implemented to provide resources that helped the interaction between phases.

For the success of research, the processes and techniques must be fluid and applied in a direction based on good guides and recommendations. The research must go through phases, with well-established bases and tools suitable for its purpose, using sources and instruments available through digital strategies and systems, models, guides and feedback, and audit mechanisms.

In addition, every beginning of a new phase must be supported by well-defined pillars that encompass the exhaustive use of validations and pretests; plans for monitoring, management, and data analysis; precautions for ethical and legal issues; training of the team; and channels for effective communication.

In the broadest sense, incorporating data quality techniques and tools is analogous to going on a trip, that is, going from point A to point B. The starting point refers to good planning of issues, such as the year’s season, the quantity and type of items that will be transported, the most appropriate means of transport, the budget available, and tips and guidance available in the different means of communication. Even if the path is already known, an important step that precedes the beginning of its execution is always the definition of the best route. Consulting maps and updated conditions are always recommended since they can change over time.

However, the execution phase of a trip is not limited to reaching the final destination. During the journey, we should always be attentive to signs and directions, without obviously failing to enjoy the landscape and all its opportunities. Finally, when we arrive at our destination, we must bear in mind that to obtain the best results, it is necessary to know the best guides and tourist attractions. A wrong choice or decision can provide us with a low-quality photograph, an unexpected experience, and, as an effect, an epilogue of bad memories.

## Discussion

### Principal Findings

This study presented contributions to aid the ultimate goal of good data quality focused on findings that used some digital technology (ie, to develop a disciplined process of identifying data sources, preparing the data for use, and evaluating the value of those sources for their intended use). Key findings revealed variability and a lack of consensus in assessing data quality domains and metrics. Data quality factors included the research environment, application time, and development steps. Strategies for improving data quality involved using business intelligence models, statistical analyses, data mining techniques, and qualitative approaches. The findings highlight the need for standardized practices and collaborative efforts to enhance data quality in health research.

The routine of health services that deal with demands for collecting and consuming data and information can benefit from the set of evidence on tools, processes, and evaluation techniques presented here. Increasingly ubiquitous in the daily lives of professionals, managers, and patients, technology should not be adopted without a specific purpose, as doing so can generate misinterpreted information obtained from unreliable digital health devices and systems. The resources presented can help guide medical decisions that not only involve medical professionals but also indirectly contribute to avoiding decisions based on low-quality information that can put patients’ lives at risk.

With the promotion of the data culture increasingly present in a transversal way, research and researchers can offer increasingly more reliable evidence and, in this way, benefit the promotion and approach to the health area. This mutual cycle must be transparent so that there is awareness that adherence to such a practice can favor the potential strengthening of a collaborative network based on results and promote fluidity and methodological transparency. In addition, it encourages data sharing and, consequently, the reuse of data into reliable information silos, enhancing the development and credibility of health research. At the international level, platforms with a centralized structure of reliable data repositories of patient records that offer data sharing have reduced duplication of efforts and costs. This collaboration can further decrease disparate inequities between middle- and high-income, giving celerity and minimizing risks in the development and integrity of studies.

Reliable data can play a crucial role in enlightening health institutions that prioritize cultivating a data-centric culture and are well equipped to deliver high-quality information. This, in turn, facilitates improved conditions for patient care. In addition to mapping concepts between different sources and application scenarios, it is essential to understand how initial data quality approaches are anchored in previous concepts and domains, with significant attention to suitability for use, following guidelines or using frameworks in a given context [41]. Since the concept in the same data source can change over time, it is still necessary to carry out mapping with an emphasis on its dimensions in a sensible way and on how the evolution of concepts, processes, and tools impacts the quality assessment of research and health services [47].

The realization of mapping with emphasis on domains or concepts must coexist in health information systems. The outcome favors maximizing processes, increasing productivity, reducing costs, and meeting research needs [26]. Consequently, within legal and ethical limits, it is increasingly necessary to use data comprehensively and efficiently to benefit patients [57]. For example, recent clinical and health service research has adopted the “fit for use” concept proposed in the information science literature. This concept implies that data quality dimensions do not have objective definitions but depend on tasks characterized by research methods and processes [48]. Increasingly, data quality research has borrowed concepts from various referencing disciplines. More importantly, with many different referencing disciplines using data quality as a context within their discipline, the identity of the field of research has become increasingly less distinct [33].

### Comparison With Prior Work

The large dissonance between domain definitions has increasingly motivated the search for a gold standard to be followed [30]. The area has received particular attention, especially after the term “big data” gained increasing strength [58]. The human inability to act with a large volume of information in research and the need to control this high data volume are increasingly driving the emergence of digital solutions. Although the definition of these digital data quality tools occurs from the end user’s perspective, their implementation occurs from the researcher’s perspective; a data set is highly context specific [33]. So, a generic assessment framework is unlikely to provide a comprehensive data quality analysis for a specific study, making its selection dependent on the study’s analysis plan [40].

The use of ontologies, for example, can help quantify the impact of likely problems, promote the validity of an effective electronic measure, and allow a generalization of the assessment approach to other data analysis tasks in more specific domains [55]. This benefit allows the decision-making process and planning of corrective actions and resource allocation faster [47]. However, the complex coding process can generate inconsistencies and incompleteness due to the characterization of clinically significant conditions, insufficient clinical documentation, and variability in interpretation [30]. Therefore, it is critical to use specific rules that capture relevant associations in their corresponding information groups. Administrative health data can also capture valuable information about such difficulties using standardized terminologies and monitor and compare coded data between institutions [24].

Nevertheless, as a consequence of this lack of standard, the use of integrated quality assurance methods combined with standard operating procedures (SOPs) [58], the use of rapid data feedback [38], and supportive supervision during the implementation of surveys are feasible, effective, and necessary to ensure high-quality data [31]. Adopting such well-defined interventions still plays an essential role in data quality management. It is possible to perform these activities through process control and monitoring methods, data manipulation and visualization tools, techniques, and analysis to discover patterns and perspectives on the target information subset [27]. Regardless of the model adopted, these tools should aim to discover abnormalities and provide the ability to stop and correct them in an acceptable time, also allowing for the investigation of the cause of the problem [56].

Technology is an excellent ally in these processes, and in parallel with the tools of the Lean Six Sigma philosophy, it can partially replace human work [31]. To maximize the potential of this combination, the value derived from using analytics must dictate data quality requirements. Computer vision/deep learning, a technology to visualize multidimensional data, has demonstrated data quality checks with a systematic approach to guarantee a reliable and viable developed asset for health care organizations for the holistic implementation of machine learning processes [53]. However, most of these analytical tools still assume that the analyzed data have high intrinsic quality, which can thus allow possible failures in the process, in addition to the final experiments’ lack of optimization, safety, and reliability [37].

In this way, the reuse of information has a tremendous negative impact [48]. The centralized storage of variables without excellent mapping to changes in system paradigms (metadata) and with a mechanism to trace the effects of changes in concepts that are frequent in the health area can also affect the reliability of research [37]. For example, the severity classification of a given condition can change over time and, consequently, mitigate the comparability power of a study or even prevent it from being used as a basis for planning or evaluating a new one [52]. In addition, the cultural background and experience of researchers can influence the interpretation of data [44]. Therefore, a combination of integrated tools located centrally and at each partner site for decentralized research networks can increase the quality of research data [40].

A central metadata repository contains common data elements and value definitions used to validate the content of data warehouses operated at each location [34]. So, the consortium can work with standardized reports on data quality, preserving the autonomy of each partner site and allowing individual centers to improve data in their locally sourced systems [29]. It is, therefore, essential to consider the quality of a record’s content, the data quality usability, and what mechanisms can make data available for broader use [41]. As outlined by Kodra et al [42], managing data at the source and applying the FAIR guiding principles for data management are recognized as fundamental strategies in interdisciplinary research network collaboration.

Data production and quality information dissemination depend on establishing a record governance model; identifying the correct data sources; specifying data elements, case report forms, and standardization; and building an IT infrastructure per agreed principles [29]. Developing adequate documentation, training staff, and providing audit data quality are also essential and can serve as a reference for teaching material for health service education [25]. This can facilitate more quality studies in low- and middle-income countries.

The lack of such studies implies that health systems and research performance in these countries still face significant challenges at strategic stages, such as planning and managing complete data, leading to errors in population health management and clinical care [43]. In turn, the low use of health information and poor management of health information systems in these countries make evidence-based decisions and planning at the community level difficult [2]. The results also demonstrate that, despite existing, such individual training efforts focus mainly on transmitting data analysis skills [33].

### Strengths

Identifying systematic and persistent defects in advance and correctly directing human, technical, and financial resources are essential to promote better management and increase the quality of information and results achieved in research [42]. This step can provide improvements and benefits to health managers, allowing greater efficiency in services and better allocation of resources. Promoting such benefits to society through relevant data impacts the performance and effectiveness of public health services [39] and boosts areas of research, innovation, and enterprise development [59].

Creative approaches to decision-making in data quality and usability require good use of transdisciplinary collaboration among experts from various fields regardless of study design planning or application area [59]. This use may be reaching the threshold of significant growth and thus forcing the need for a metamorphosis from the measurement and evaluation of data quality, today focused on content, to a direction focused on use and context [57].

Without a standard definition, the use of the “fit for purpose” concept for performance monitoring, program management, and data quality decision-making is growing. As a large part of this quality depends on the collection stage, interventions must target the local level where it occurs and must encompass professionals at the operational level and forms at the technical level. Identifying and addressing behavioral and organizational challenges and building technical capacity are critical [60], increasingly fostering a data-driven culture [29,30].

### Limitations

Among the limitations of our review, we first highlight the search for works written in English and Portuguese, since the interpretation of concepts and even the literal translations of terms referring to the dimensions and adaptations to different cultural realities can vary, and thus influenced part of our evaluation [31]. The limitation may impact the results by excluding relevant research published in other languages and overlooking diverse cultural perspectives. To mitigate this, we suggest expanding collaboration with multilingual experts and including studies in various languages to ensure a comprehensive and unbiased evaluation of data quality.

Second, the absence of evidence in middle-income countries prevented the authors from conducting an adequate synthesis regarding the performance and application of the evidence found in these countries [2]. Limited representation from middle-income countries hinders the generalizability and applicability of findings, risking a biased understanding of intervention effectiveness. Inclusion of more studies from middle-income countries is vital for comprehensive evidence synthesis, enabling better comprehension of intervention performance in worldwide contexts and avoiding oversight of critical perspectives and outcome variations.

Third, due to the rapid growth of technologies applied to the area, we conducted a search focused on the past 5 years, which may draw attention away from other fundamentals and relevant procedures. The limited time span may lead to incomplete findings and conclusions, hindering a comprehensive understanding of the field’s knowledge and advancements. To address this limitation, future research should consider a broader time frame to include older studies, allowing for a more thorough examination of fundamentals and relevant procedures impacted by the rapid evolution of technologies in the area.

### Future Directions

Once the technical and organizational barriers have been overcome, with data managed, reused, stored, extracted, and appropriately distributed [46], health care must also pay attention to behavior focused on interactions between human, artificial, and hybrid actors. This interaction reflects the importance of adhering to social, ethical, and professional norms, including demands related to justice, responsibility, and transparency [60]. In short, increasing dependence on quality information increases its possibilities [61], but it also presents regulators and policy makers with considerable challenges related to their governance in health.

For future work, developing a toolkit based on process indicators is desirable to verify the quality of existing records and provide a score and feedback on the aspects of the registry that require improvements. There is a need for coordination between undergoing initiatives at national and international levels. At the national level, we recommend developing a centralized, public, national “registration as a service” platform, which will guarantee access to highly trained personnel on all topics mentioned in this paper, promoting the standardization of registries. In addition to allowing cost and time savings in creating new registries, the strategy should allow for linking essential data sources on different diseases and increase the capacity to develop cooperation at the regional level.

We also suggest using the data models found in this study to serve as a structured information base for decision support information system development and health observatories, which are increasingly relevant to public health. Furthermore, concerning the health context, it may allow the execution of implementation research projects and the combination with frameworks that relate to health behavior interventions, for example, the Reach, Effectiveness, Adoption, Implementation, and Maintenance (RE-AIM) framework [62], among others.

### Conclusion

This study will help researchers, data managers, auditors, and systems engineers think about the conception, monitoring, tools, and methodologies used to design, execute, and evaluate their research and proposals concerned with data quality. With a well-established and validated data quality workflow for health care, it is expected to assist in mapping the management processes of health care research and promote the identification of gaps in the collection flow where any necessary data quality intervention can be accordingly evaluated with the best tools described here. In conclusion, the results provide evidence of the best practices using data quality approaches involving many other stakeholders, not just researchers and research networks. Although there are some well-known data quality guidelines, they are context specific and not found in the identified scientific publications. So, the information collected in this study can support better decision-making in the area and provide insights that are distinct from the context-specific information typically found in scientific publications.

## Appendix

Multimedia Appendix 1 Preferred Reporting Items for Systematic Reviews and Meta-Analyses (PRISMA) checklist.

Multimedia Appendix 2 Individual studies.

## Abbreviations

AI: artificial intelligence
CINAHL: Cumulative Index of Nursing and Allied Health Literature
IEEE: Institute of Electrical and Electronics Engineers
LILACS: Latin American and Caribbean Health Sciences Literature
PRISMA: Preferred Reporting Items for Systematic Reviews and Meta-Analyses

## Data Availability

The data sets generated and analyzed during this study are available as Multimedia Appendix 2 or can be obtained from the corresponding author upon reasonable request.
